# Robotic abdominoperineal resection, posterior vaginectomy and abdomino-lithotomy sacrectomy: technical considerations and case vignette

**DOI:** 10.1007/s10151-023-02827-w

**Published:** 2023-07-15

**Authors:** C. C. Kearsey, M. Mathur, P. A. Sutton, C. R. Selvasekar

**Affiliations:** 1 Colorectal and Peritoneal Oncology Centre, The Christie NHS Foundation Trust, Wilmslow Rd, Manchester, M20 4BX UK; 2 Institute of Translational Medicine, University of Liverpool, Liverpool, UK; 3 Lancashire Teaching Hospitals NHS Foundation Trust, Preston, UK; 4 Division of Cancer Sciences, University of Manchester, Manchester, UK; 5 Faculty of Health, Social Care and Medicine, Edge Hill University, Ormskirk, UK

**Keywords:** Pelvic exenteration, Robotic surgery, Rectal cancer

## Abstract

When working with patients who have locally advanced rectal cancer (LARC) the ability to undertake minimally invasive procedures becomes more challenging but no less important for patient outcomes. We performed a minimally invasive approach to surgery for LARC invading the posterior vagina and sacrum. The patient was a 75-year-old lady who presented with a locally advanced rectal tumour staged T4N2 with invasion into the posterior wall of the vagina and coccyx/distal sacrum. We introduce a robotic abdominoperineal resection, posterior vaginectomy and abdomino-lithotomy sacrectomy using a purely perineal approach with no robotic adjuncts or intracorporal techniques. Final histology showed moderately differentiated adenocarcinoma invading the vagina and sacrum, ypT4b N0 TRG2 R0 and the patient entered surgical follow-up with no immediate intra- or postoperative complications. A literature review shows the need for more minimally invasive techniques when relating to major pelvic surgery and the benefits of a purely perineal approach include less expensive resource use, fewer training requirements and the ability to utilise this technique in centres that are not robotically equipped.

## Introduction

In the past two decades, the most significant change in medical practice has been the reduction of surgical trauma. New surgical or interventional techniques primarily focus on a minimally invasive approach, which is now widely adopted. The goal of minimal access therapy is to minimize the damage caused by the procedure while ensuring the treatment’s safety and effectiveness compared to traditional open surgery. To achieve this, high-quality image display systems and intraoperative technology are crucial, along with team training and the expertise of the surgeon, enabling precise and personalized surgery. The ultimate objective is to enable patients to recover faster, resulting in shorter hospital stays and a quicker return to normal activities and work [1].

When dealing with patients diagnosed with locally advanced rectal cancer (LARC), performing minimally invasive procedures becomes more challenging yet equally essential for favourable patient outcomes. Here, we present an approach that utilizes minimally invasive techniques for surgery involving LARC that has spread to the posterior vagina and sacrum.

## Methods

### Patient

**Fig. 1:**
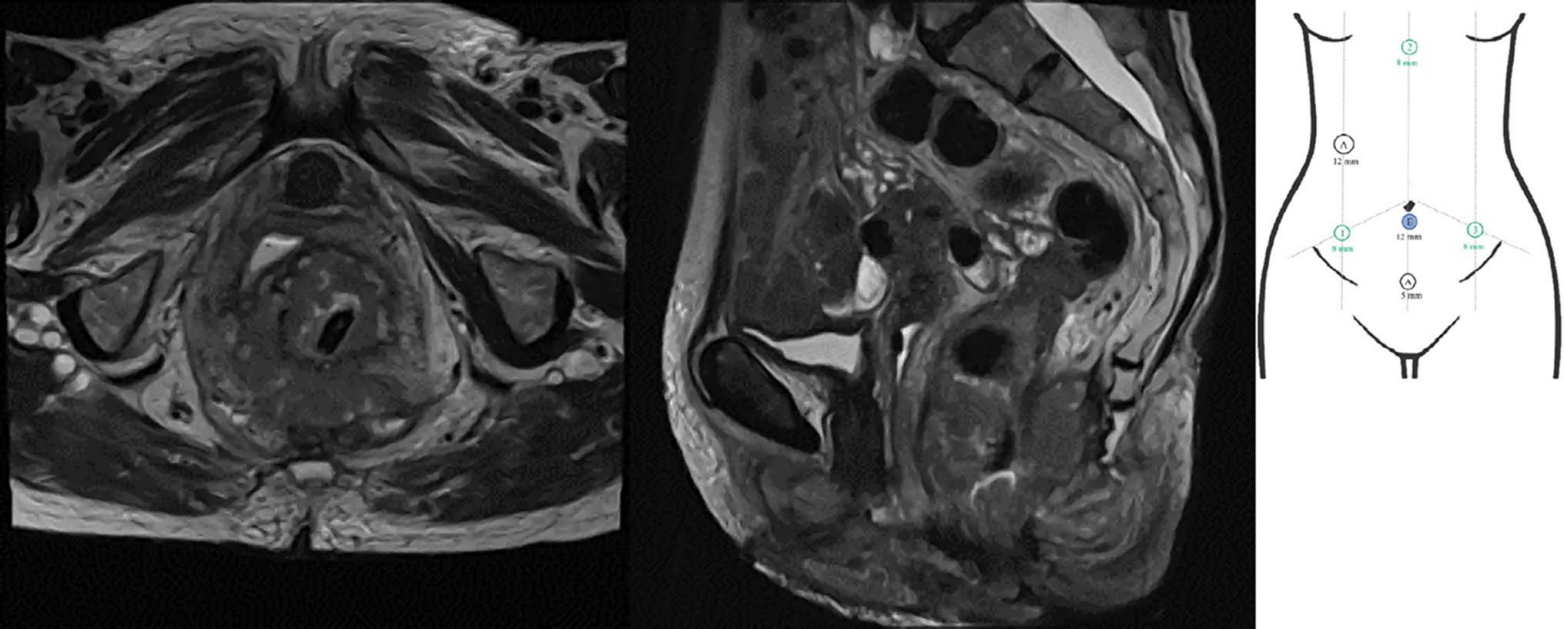
T2 weighted axial (left) and sagittal (right) sections of the MRI that clearly show the rectal tumour involving S5 and posterior vagina.  Figure 2 Diagrammatic representation of port placement

A 75-year-old lady presented with a locally advanced rectal tumour staged T4N2 with invasion into the posterior wall of the vagina and coccyx/distal sacrum. She underwent a loop colostomy and neoadjuvant chemoradiotherapy followed by consolidation chemotherapy (total neoadjuvant therapy). There was a partial response to treatment, although the tumour remained in contact with the coccyx/lower sacrum posteriorly, tethering the vagina anteriorly. As can be seen in Fig. 1, the predominant invasion is of the coccyx with an area of restricted diffusion extending to S5 which was suspicious for viable tumour. In the opinion of the multidisciplinary team (MDT), coccygectomy alone would have risked a positive margin. Following MDT discussion, it was decided that the patient would be offered robotic abdominoperineal resection with en bloc posterior vaginectomy and distal (S5) sacrectomy.

### Operative technique and follow-up

The procedure was undertaken on the Intuitive da Vinci Si™ platform (Intuitive Surgical, Sunnyvale, CA, USA) Standard four-arm docking technique used for robotic abdominoperineal resections was employed with the robot docking over the patient’s left hip in a standard fashion. Figure 1 shows port placement.

A medial to lateral approach was adopted following division of the loop colostomy. The inferior mesenteric artery was identified and divided. Circumferential dissection of the total mesorectal excision (TME) was undertaken to the level of tumour involvement at the sacrum and vagina. A transverse vaginotomy was created in the posterior wall of the vagina as the final part of the abdominal phase of the procedure to assist the perineal phase of the dissection. Laterally the dissection was taken down to the pelvic floor on both sides. The abdominal component of the operation was now complete. The colostomy was refashioned in the left iliac fossa and the abdominal ports closed.

**Fig. 2:**
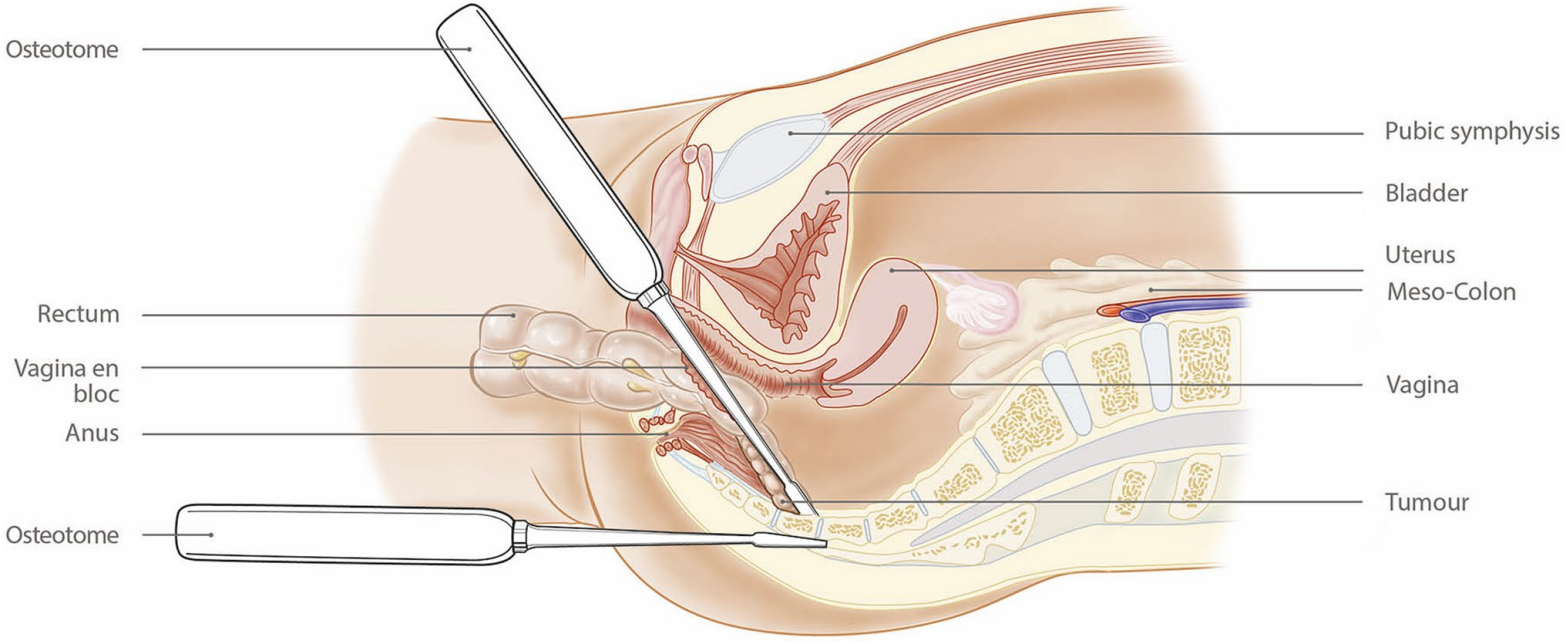
Diagrammatic sagittal section showing the everted rectum delivered through the perineal wound with the tumour dissected from the posterior vagina and pelvic side

**Fig. 3:**
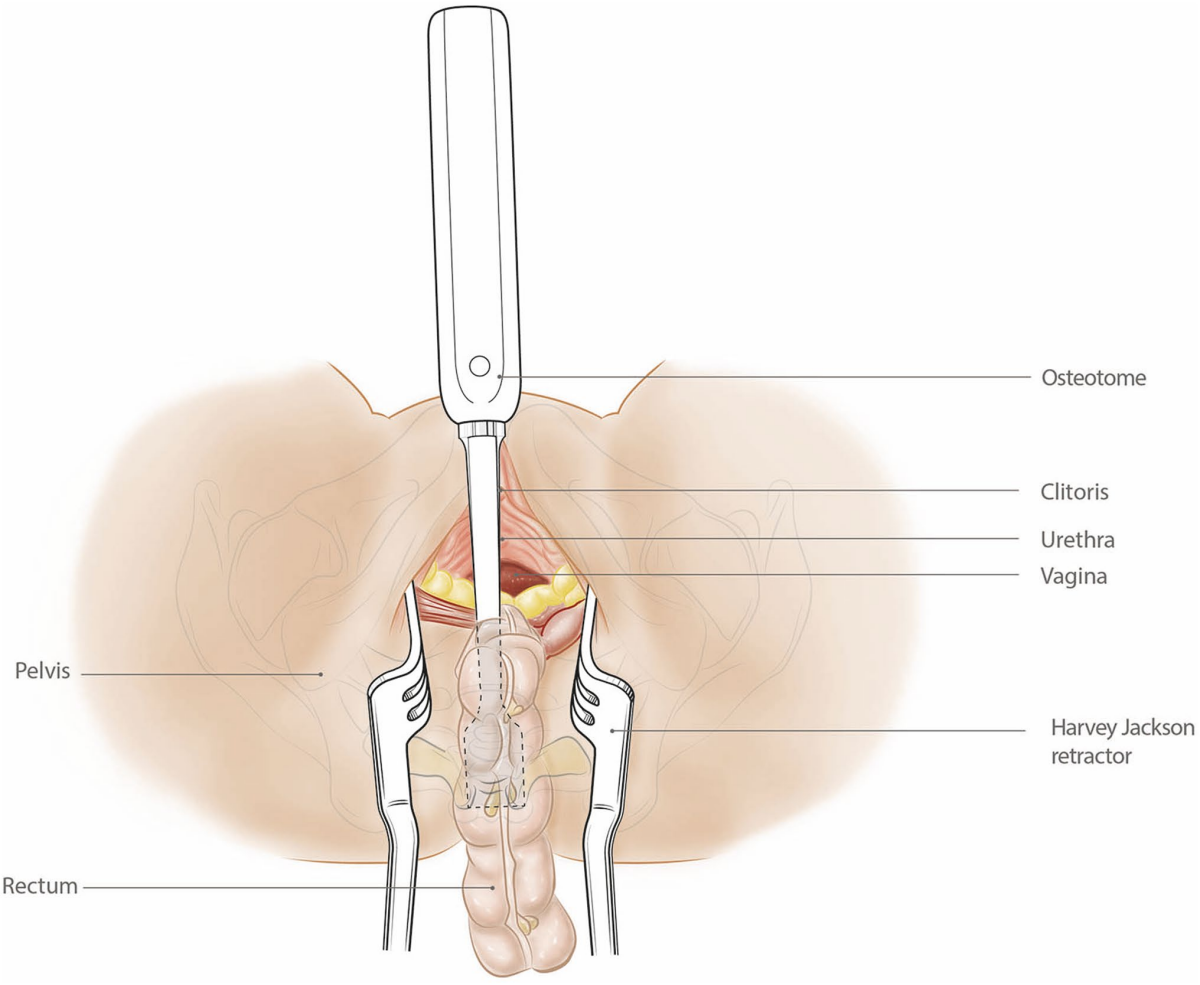
Diagrammatic view of Fig. 2 from the perineal viewpoint with the osteotome in position

The perineal phase involved dissection in the extra-levator plane, initially leaving the anterior and posterior dissection planes. The pelvic cavity was entered laterally on both sides through the levator plate, and the dissection was continued anteriorly to meet the vaginotomy created during the abdominal phase of the procedure. The vagina was divided with an energy device along its length to excise the posterior wall en bloc with the specimen. At this point, the only remaining attachment was the distal sacrum at S5. The perineal dissection was continued posteriorly in the post-sacral space along the posterior and lateral aspects of the sacrum beyond the level of invasion, ensuring that this dissection met with the laterally divided levator to leave a clearly exposed bony edge. The specimen was everted and delivered through the perineal wound. A long handheld osteotome was placed in the post-sacral space, and a second osteotome placed through the perineal wound to divide the sacrum at the S5 level (Figs. 2, 3). The perineal defect was closed with a local advancement (V–Y) flap.

The total operating time was 6 h with approximately 100 ml of intraoperative blood loss. There were no postoperative complications and the colostomy functioned on day 3 postoperatively.  The length of hospital stay was 10 days. Follow-up by plastic surgery and colorectal teams at 4–6 weeks revealed no complications. Final histology showed moderately differentiated adenocarcinoma invading the vagina and sacrum, ypT4b N0 TRG2 R0 and the patient entered surgical follow-up.

## Discussion

Pelvic exenteration, initially described by Brunschwig in 1948, and later applied to colorectal cancer by Butcher and Spjut in 1959 [2], has historically been associated with elevated rates of complications and mortality. These challenges stem from the technical complexities involved in removing multiple organs within the limited space of the pelvis [3]. In cases of LARC, where the tumour extends beyond the mesorectal envelope, achieving clear margins often necessitates a multivisceral resection beyond the traditional surgical plane of TME [4]. Approximately 6–10% of patients with rectal cancer have tumours involving adjacent organs at the time of diagnosis, making en bloc excision of both the tumour and adjacent organs beneficial [7]. While originally intended as a palliative procedure, pelvic exenteration has undergone significant advancements. The 5-year overall survival rate for patients with advanced pelvic malignancies who undergo pelvic exenteration now ranges from 22% to 66% [5], compared to less than 5% for non-surgical management options [6]. This improvement in long-term outcomes can be attributed to enhanced perioperative care and the development of improved surgical techniques, particularly the utilization of minimally invasive approaches like laparoscopic surgery. While laparoscopic surgery reduces intraoperative blood loss, enables faster recovery rates, faster return of bowel function, and shorter hospital stays in standard TME surgery [7], it has technical limitations when performing complex pelvic dissections beyond the scope of TME. Consequently, there is a pressing need for a more ergonomically and visually enhanced minimally invasive approach. Robot-assisted technologies have made significant strides in the treatment of LARC and multivisceral pelvic exenteration beyond the TME plane, where confined spaces pose operative challenges. In 2014, Nanayakkara et al. reported the first robot-assisted pelvic exenteration for LARC using the da Vinci^®^ surgical system [7]. Since then, there has been a gradual increase in the number of case reports and series worldwide, demonstrating the safety and feasibility of robot-assisted multivisceral resections for locally advanced and recurrent rectal cancers [8–14]. Various generations of the da Vinci robots have been employed, with comparable operative times, blood loss, and achievement of complete oncological resection margins. Conversion rates to open surgery were low, with only one reported case. Postoperative complication rates were generally low in most case series, though larger-volume case series observed higher rates, reflecting the inherent morbidity of multivisceral pelvic exenteration.

Like all patients with colorectal cancer, those with LARC must undergo an extensive MDT process. Patient selection plays a crucial role in achieving favourable oncological outcomes. Initially, involvement of the sacrum in exenteration surgery was considered unsuitable for curative treatment. However, specialist centres now routinely perform en bloc sacral resection to ensure clear resection margins, with acceptable morbidity, oncological results, and functional outcomes [15]. Sacrectomy, a component of pelvic exenteration, can be categorized as high (above the junction of S2–S3) or low (below the S2–S3 junction) on the basis of the location of the disease. Higher sacral resections can pose technical challenges and result in significant postoperative morbidity and functional impairment due to nerve root sacrifice. These factors have discouraged many surgeons from performing high sacrectomy, particularly when the measurable survival benefit remains unclear [16–18].

In 2015 a technique was described which in select cases negates the need for high sacrectomy. The high subcortical sacrectomy (HiSS) technique was developed in St Marks Hospital and involves resection of the anterior cortex and cancellous bone of the sacrum whilst maintaining the stability of the pelvic girdle and avoiding the surgical morbidity associated with high sacrectomy. It is important to consider, however, that the immediate complications associated with such major resections remain, and careful patient selection and surgeon experience are essential to perform these procedures [19]. This technique of abdomino-lithotomy sacrectomy would be below the S2–S3 level.

Robotic sacrectomy has been described once before but using a specialised ultrasonic bone divider which is an adjunct of the da Vinci™ robotic system. The short-term outcomes using this intracorporeal method to resect the involved sacrum were comparable to our perineal resection [20]. The disadvantage of using robotic adjuncts, apart from the cost implications, is the need for additional training that would be required to use such equipment. The procedure described in our centre provides the opportunity to convert this procedure to a laparoscopic operation, further reducing the cost and opening this technique up to other centres/surgeons that may not have robotic capabilities. One specific disadvantage of the technique that we have described here is its limited suitability to only very distal sacrectomies (S4/S5) due to challenges that would arise from osteotome placement through the perineal wound. Simulation training and cadaveric courses would be of high value in disseminating this procedure nationally and this is in the process of being introduced.

Negative resection margins (R0) are the single most important prognostic factor in predicting long-term survival and quality of life in patients undergoing pelvic exenteration [1, 5, 7, 8, 13] and the goal of exenterative surgery is to resect all involved organs/structures whilst balancing this radicality with an acceptable risk profile and postoperative quality of life. One clear benefit of our procedure is the minimally invasive nature of the technique, promoting enhanced patient experience whilst still achieving an R0 resection.

When selecting patients for this approach, good communication between surgical and radiological colleagues in a well-constructed MDT is critical for the accurate assessment of resectability. Expertise from multiple specialities is required to devise a radiological/surgical plan that highlights potential issues and ensures negative margins. One of the most important aspects of tailoring treatment is focusing the planning of the resection on the maximum possible disease extent identified on MRI imaging, regardless of down-staging post neoadjuvant treatment. It can be seen in our case that there was a moderate reduction in post-treatment tumour volume in subsequent MRI scans; however, the need for S5 sacrectomy was still deemed essential for negative margins. Occult microscopic foci of viable tumour cells can be harboured within any fibrosis remaining on MRI and should be considered to have malignant potential. The final histological analysis following this approach confirmed that resection of the sacrum was necessary for clear margins, despite a reasonable response to adjuvant treatment.

With appropriate patient selection and surgical team experience this procedure offers a minimally invasive option for sacrectomy with low (S4–S5) sacral involvement. This technique of abdomino-perineal lithotomy sacrectomy has been performed five times prior to this in an open fashion, and since this operation was undertaken another three procedures have been successfully carried out with no intra- or postoperative complications. A larger series is needed to further analyse the short- and long-term complications.

## Data Availability

The data referred to in this paper is available on request from the corresponding author, but is not public due to privacy restrictions.
